# Individual, Social and Environmental Correlates of Active School Travel among Adolescents in India

**DOI:** 10.3390/ijerph17207496

**Published:** 2020-10-15

**Authors:** Abishamala Kingsly, Anna Timperio, Jenny Veitch, Jo Salmon, Rajendra Pradeepa, Harish Ranjani, Ranjit Mohan Anjana

**Affiliations:** 1 School of Exercise and Nutrition Sciences, Institute for Physical Activity and Nutrition (IPAN), Formerly, Deakin University, Geelong 3220, Australia; abisha.91k@gmail.com (A.K.); anna.timperio@deakin.edu.au (A.T.); jo.salmon@deakin.edu.au (J.S.); 2 Madras Diabetes Research Foundation & Dr.Mohan’s Diabetes Specialities Centre, WHO Collaborating Centre for Non-Communicable Diseases & ICMR Centre for Advanced Research on Diabetes, Chennai 600086, India; rguhapradeepa@gmail.com (R.P.); ranjani@mdrf.in (H.R.); dranjana@drmohans.com (R.M.A.)

**Keywords:** active school travel, active transport, barriers, correlates, physical activity, neighbourhood environment, adolescents, India

## Abstract

Active School Travel (AST) is an important domain for physical activity among adolescents; however, few studies in India have explored barriers or correlates of AST. This was a cross-sectional study of 324 adolescents aged 12–17 years recruited via households and schools from diverse areas of Chennai, India. Adolescents reported their mode of travel to school, neighbourhood correlates, and the barriers for AST. Adolescents were considered to be using AST to/from school if they walked/cycled ≥once/week during an average week. Half the adolescents usually performed AST (≥1 trip/week). School being too far was associated with 75% lower odds and parents not allowing their child to walk or cycle was associated with 82% lower odds of the adolescent performing AST to or from school at least once/week. AST among adolescents should be encouraged and there is considerable scope for improvement. Parental restriction and distance to school were the two strongest barriers for AST.

## 1. Introduction

Identifying ways to increase physical activity through all ecological domains (e.g., active school travel, outdoor physical activity, leisure-time physical activity) of everyday life has become imperative to increasing overall levels of physical activity [1,2,3]. Globally, studies have identified Active School Travel (AST) (i.e., walking or cycling to school) as one way in which adolescents can increase their physical activity levels [4,5]. In accordance with social ecological models [6], individual, social, family and environmental factors all play a role in influencing engagement in AST [7,8].

Individual factors such as age, gender and positive attitudes have been shown to have a positive influence on AST among children and adolescents. Similarly, social support has been reported by studies from the US and Australia to be an important social factor related to AST [9]. Urbanization and features of the neighbourhood built environment such as urban sprawl have increased the need to use motorized transportation modes, thereby decreasing adolescents’ AST [10,11]. Walking and cycling for transport has declined due to safety concerns both among adolescents and their parents [7,8]. Location of schools further from home, land use mix, residential density, crime and traffic safety have all been associated with active travel in children or youth [7,12,13]. A wide breadth of research has been conducted globally that reinforces the influence of factors from multiple levels of socioecological models on AST in children and adolescents; however, research among adolescents in Asian countries is less common [14].

In India, millions of children and youth travel to school every day [15], and escorting adolescents to school via a range of motorized travel modes is common [16]. In the 2018 India Report Card on physical activity for children and youth, a grade of B- was assigned for active transportation (weighted average of ~65% regularly walking or cycling to school) [17]. Studies among adolescents show varying rates of AST among adolescents by region, ranging from 23% among 11–15 year olds in one school in Coimbatore [18], 57% for usual walking and 6% for cycling among 11–14-year-olds in Hyderabad [16], 65% walking or cycling to school every day in Chennai [19], and 74% of 10–14-year-olds using AST in rural areas of Chennai, Goa and Matlab (Bangladesh) [20]. While there are some data on rates of AST, few studies have explored barriers and facilitators of AST among adolescents in India, especially across all levels of social ecological models. Aspects of the built environment have been found to be associated with adults use of active transport to work in Chennai [21], but are rarely explored among adolescents. Tetali et al. [16] reported that distance was inversely related to AST and that youth attending private schools (high household income) were less likely to use an active travel mode but did not explore other barriers and facilitators in the individual, social or built environment. This is not surprising, as in India sedentary modes of transport (i.e., car) are considered a symbol of social status and are more affordable for the high household income strata [16]. Thus, there is a need to study more comprehensive correlates of AST covering all levels of the socioecological model. The aim of this study was to understand the correlates of adolescents’ AST in India. The findings will help inform the development of future strategies to improve rates of AST in India.

## 2. Materials and Methods

### 2.1. Study Setting and Sampling Procedure

Data were collected from a cross-sectional survey among 324 adolescents and their parents. The study was conducted in the largest metropolis of southern India, Chennai. This sample size was computed to be sufficient to detect a 40% prevalence of meeting physical activity guidelines based on earlier studies [22], with 5% precision, 80% power and a design effect of 2. Data were collected using convenience sampling between February 2015 and December 2015. Adolescents aged 12–17 years were recruited from 157 of the 200 wards (smaller administrative units), covering all 15 zones (larger administrative units) of the city. To be eligible for inclusion in the study, adolescents had to be12–17 years of age; willing to complete the survey (both parents and adolescents) and the distance between each participating adolescent’s residence needed to be at least 500 m to maintain diversity in neighbourhood environments.

Adolescents were recruited via private schools that agreed to participate in the study as well as “door-to-door” recruitment, approaching known contacts from previous studies conducted by the Madras Diabetes Research Foundation (MDRF), contacts from the staff of the MDRF and cold calling telephone numbers from the Chennai telephone directory, were used. Of these 324 participants, 65% were recruited via non-school recruitment method (door-to-door = 201 participants; MDRF network = 10 participants) and 35% (*n* = 113) through schools. Higher proportions of those recruited via schools had high household income compared to those recruited through non-school avenues (26% vs. 11%). Adolescents from different schools had different school start and finish times and different neighbourhood environments. All parents gave their informed consent and assent from the adolescents was obtained before they participated in the study. The study was approved by the Institutional Ethics Committee of the Madras Diabetes Research Foundation, Chennai, Tamil Nadu, India and the Deakin University Human Research Ethics Committee, Australia. Prior to the start of the study written informed consent from parents and assent from adolescents for inclusion was obtained.

### 2.2. Measures and Variables

Adolescent–parent pairs completed surveys based largely on the NEWS and NEWS-Y instruments [23,24].

#### 2.2.1. Dependent Variables

Active school travel and other modes of travel: Adolescents were asked ‘In an average school week, on how many days do you use the following modes of transport to get to and from school?’ [25]. Options included: walk, bicycle (self), skateboard, auto rickshaw, cycle rickshaw, school bus/private van, two-wheeler (pillion rider of a bicycle or a motorbike/self-driving of a motorbike), car and public transport. Based on modal use at least once per week, the following variables were created (never vs. ≥once/week): walk to/from school, cycle (self) to/from school, public transport to/from school, car to/from school, skate board to/from school, other assisted travel mode to/from school and AST to/from school. Assisted travel mode included travel when accompanied by a parent, friend or other traveller (e.g., cycle rickshaws, auto rickshaws, school van, car and using a two-wheeler). AST included walking or cycling to and from school, which were combined and dichotomised into a single item to compute AST (no AST vs. AST ≥ 1 trip/week) as the data were zero-inflated.

#### 2.2.2. Independent Variables

Perceived barriers to AST: Perceived barriers specifically to AST were reported by adolescents. The items were adapted from previous research [26]. Adolescents were asked their level of agreement with seven statements classified as personal-level barriers (e.g., “I am too lazy to walk or cycle to school”), seven statements classified as social/family level barriers (e.g., “My parents do not allow me to walk/cycle to school”) and 11 statements classified as environmental-level barriers (e.g., “There are too many hills”). Items added for local relevance included open drains on the road, road closures for renovation and potholes/stagnant water. Response options included: (1) strongly disagree, (2) somewhat disagree, (3) somewhat agree, and (4) strongly agree. The responses were collapsed into two categories and coded as: (0) strongly disagree/somewhat disagree; (1) somewhat agree/strongly agree.

Crime safety score: Adolescents and parents reported on perceived crime, based on two items each from the NEWS-Y and NEWS, respectively [23,24] (e.g., “High crime rate in my neighbourhood, crime rate makes it difficult to go on walks”). Response options included: (1) strongly disagree, (2) somewhat disagree, (3) somewhat agree, and (4) strongly agree. All individual items were reverse-coded and averaged to compute an adolescent (Cronbach’s alpha= 0.64) and a parent-report score (Cronbach’s alpha= 0.66). Higher scores indicated a more favourable view (e.g., a higher crime safety score portraying a better perception of safety).

Stranger danger score [23]: A score was computed for adolescents based on the mean of responses to six items related to feeling threatened by groups of people/gangs and being worried about certain situations because they are afraid they might be taken or hurt by a stranger. The questions were the same as NEWS-Y, but the scoring procedure for the score “stranger danger” was as reported by Parker et al. [25]. Response options included: (1) strongly disagree, (2) somewhat disagree, (3) somewhat agree, and (4) strongly agree. A similar mean score was also computed for parents based on four items. All items were reverse coded prior to computing the scores (adolescent Cronbach’s alpha = 0.83 and parent Cronbach’s alpha = 0.88). A higher score indicated more favourable conditions for physical activity (i.e., lower stranger danger).

Land use mix–diversity: Responses to 32 parent-reported items assessing presence of destinations within specified distances from home were coded as follows: 1–5 min (1); 6–10 min (2); 11–20 min (3); 21–30 min (4); 31+ min (5) and do not know or not present (8). Consistent with scoring protocols used in NEWS–Y [23], which was the primary source of the items used in this study, responses of 31+ mins and do not know or not present were combined for each destination during data analysis, as both responses indicate destinations were mostly inaccessible by walking. Destinations included recreation facilities, shops, eateries and other locations. All items were reverse-coded, and the mean of all items was calculated to create a mean access score (possible range 1–5). Higher scores indicated a more favourable environment for walking in terms of diversity of destinations.

Public recreation spaces: A mean access score was created based on five items assessing access to public recreation spaces within specified distances from home including: small public parks, large public parks, cycling/walking trails, “beach, ponds or lakes” and public open spaces. The items were coded as follows: 1–5 min (1); 6–10 min (2); 11–20 min (3); 21–30 min (4); 31+ min, and do not know or not present (5). All items were reverse-coded prior to creating the score based on adolescent and parent-report. Higher scores indicated higher access.

Land use-mix access [24]: Parents responded to three items regarding access to land-use mix (stores are at a walkable distance from their homes’, “presence of recreational facilities”, and “it is easy for teenagers to walk to transit stops from their home”). Response options included: (1) strongly disagree, (2) somewhat disagree, (3) somewhat agree, and (4) strongly agree. Average scores were created with higher scores denoting better perceived access to services (Cronbach’s alpha = 0.35).

India-specific barriers/obstruction to walking: Parents were asked how much they agreed with nine statements about barriers to walking in the neighbourhood including: bad roads, open drains, mosquitoes, garbage, traffic, stray dogs, cattle, odour and stagnant water. These were newly developed statements specific to local conditions in the walking environment in India. Response options included: (1) strongly disagree, (2) somewhat disagree, (3) somewhat agree, and (4) strongly agree. Items were reverse-coded and averaged to create a score, with higher scores denoting fewer barriers (Cronbach’s alpha = 0.77).

Infrastructure for walking/cycling: Parents reported on six items related to the availability and quality of pavements in their neighbourhood (e.g., presence of pavements on most of streets in neighbourhood, presence of separation between roads and pavement by parked cars). Response options included: (1) strongly disagree, (2) somewhat disagree, (3) somewhat agree, and (4) strongly agree. Responses were averaged as per the NEWS scoring protocol [27], with higher scores denoting higher walkability (Cronbach’s alpha = 0.86).

Street connectivity [23]: Parents reported on four items related to street connectivity including: distance between intersections; number of four-way intersections; presence of dead ends; and presence of multiple routes for getting to a location. Response options included: (1) strongly disagree, (2) somewhat disagree, (3) somewhat agree, and (4) strongly agree. Responses were averaged to compute a score as per the NEWS scoring protocol [27], with higher scores denoting higher walkability (Cronbach’s alpha = 0.38).

Aesthetics: Four items from the NEWS-Y [23] asked parents reported on four items related to aesthetics including: presence of shade on the pathway; having interesting things to look at; having interesting natural scenes; and having interesting buildings to look at. Response options included: (1) strongly disagree, (2) somewhat disagree, (3) somewhat agree, and (4) strongly agree. Responses were averaged to compute a score as per NEWS scoring protocol [27], with higher scores denoting higher perceived aesthetics (Cronbach’s alpha = 0.70).

Residential Density [23]: Parents were asked to report on eight items relating to residential density (e.g., “How common are detached single family residences in your neighbourhood”). Response options were on a 5-point scale: (1) None (2) A few (3) Some (4) Most and (5) All. Items were modified and adapted from NEWS [24] to include Indian type of residential units (e.g., slums, row houses). The scoring procedure was adapted from NEWS-Y [23] as follows: responses were weighted relative to single family residences, where 1–3 story residences were considered to be 11 times denser; 4–6 story residences were 25 times denser; 7–12 story residences were 50 times denser; 13–20 story residences were 75 times denser; 20 story residences were 100 times denser; row houses were 11 times denser; and slum dwellings were 125 times denser. The responses were multiplied by the weighted values and summed to create the residential density score. Higher scores denote greater residential density.

#### 2.2.3. Sociodemographic Variables

Age, gender and income: Date of birth and gender were collected as a part of the adolescent questionnaire while income of the household was collected from the parent questionnaire. Age was dichotomized into younger adolescents (12–14 years) and older adolescents (15–17 years). Income categorization was adapted from Kuppuswamy’s socioeconomic scale [28] and was collapsed into three groups for analysis: low income (earned between INR 0–Rs 11,161); middle-income (earned Rs 11,162–Rs 29,765); and high-income (earned > Rs 29,766) households.

### 2.3. Data Analysis

Data were analysed using IBM SPSS Statistics Version 24 (2016) and STATA SE 14. School travel modes are described as percentages and compared by gender, age group, and income using Chi-square or ANOVA with Scheffé post hoc tests. Associations between parents’ and adolescents’ perceived neighbourhood environment, barriers to walking/cycling to and from school and engaging in AST one or more times per week (referent category = 0 trips of AST per week) were identified using logistic regression models. In Model 1, each correlate was entered separately, adjusted for covariates including age, gender, income groups and mode of recruitment. All significant variables in Model 1 were included in Model 2 (multivariate model), along with age, gender, income groups and mode of recruitment. Both models accounted for potential clustering by school attended using the cluster command to compute robust standard errors.

## 3. Results

### 3.1. Sample Characteristics

Among the 324 adolescents, 51% were boys, 65% were 12–14 years and 34% were 15–17 years, and 34%, 44% and 21% adolescents were from low-, middle- and high-income households, respectively. The majority of respondents to the parents’ survey were mothers of the adolescents (73%) and 32% of respondents to the parent survey had a tertiary education.

### 3.2. Active Travel to and from School

Most adolescents reported that they did not usually walk to (73.5%) or from (69.1%) school, with 23.5% reporting walking to and 28.1% from school 5–6 days/week in an average week. Similarly, most adolescents reported no cycling to (78.4%) or from (78.1%) school, with 20.1% and 20.4% cycling to and from school, respectively, 5–6 days/week.

Table 1 shows that 52% of adolescents used an active mode of travel to or from school (either walking or cycling at least 1 trip/week), with no gender differences. However, a higher proportion of boys compared to girls cycled to school, and a greater proportion of girls than boys reported walking to school. A higher proportion of adolescents from low family income reported AST than those with high family income. Very few adolescents reported skateboarding (0.3%) or travelling to school by car (3.7%). After AST, the next most frequent form of transport mode by adolescents was public transport (particularly girls 34% compared to boys 18%), which included using the bus, train and share autos. Overall, 44% used assisted modes of travel, with higher use among those from high-income families.

### 3.3. Correlates of AST

The distribution of ecological correlates of adolescents’ AST is shown in Table 2, along with results of univariable (Model 1) and multivariable (Model 2) analyses predicting the odds of using AST. In model 1, at the environmental level, items such as the perceived presence of one or more dangerous crossings, school being too far away, and too much traffic were associated with lower odds of reporting AST once or more per week. At the social level, the perception that it is not fashionable to walk, having to cross unsafe places to reach places and parents not allowing their child to walk or cycle were associated with lower odds of using AST. At the individual level, lack of enjoyment of walking or cycling and being too lazy were inversely associated with AST. The India-specific barriers/obstructions to walking score was the only score that was associated with AST.

The multivariate model comprised nine variables. Of these, only two were significantly associated with AST. School being too far was associated with 75% lower odds and parents not allowing them to walk or cycle was associated with 82% lower odds of the adolescent using an active travel mode to or from school at least once/week.

## 4. Discussion

Walking or cycling to school is an important form of physical activity among children and adolescents. In both developed and developing countries, adolescents who walk or cycle to school have been identified as being more active than those who do not [4,5,16]. This study found that almost half the adolescents in this sample reported no active travel to school in a usual week. The perception that school is too far away and parents not allowing their child to walk/cycle to school were related to lower odds of AST among adolescents in Chennai, India. Identifying barriers that adolescents currently experience and neighbourhood environment features that inhibit or facilitate active school travel is a critical step in informing future interventions targeting this behaviour. Studies exploring the multiple levels of active school travel and its correlates in accordance to socioecological models is scarce in India. Hence, the present study builds on this knowledge gap.

Perceptions regarding dangerous crossings, excessive traffic, not being “fashionable” to walk/cycle, the need to walk through unsafe places, lack of enjoyment, distance, being “too lazy”, and parental restrictions were all associated with lower odds of AST in minimally adjusted models. These are consistent with individual, social and environmental levels of social ecological models. However, only distance (“too far to walk/cycle”) and parental rules (“parents do not allow”) were associated with AST in the multivariable model. It may be that safe walking, traffic, social undesirability, and enjoyment are also important, but that parental decisions mediate associations between other environmental variables and AST, particularly variables related to safety. Parental restriction, for example, may mediate associations between perceived safety and AST. This is particularly salient given that road injuries on school journeys are common among adolescents in India, particularly among cyclists [29]. Negative perceptions may also be higher the further adolescents reside from school. Numerous studies suggest that parental perceptions are highly influential [9,30]. In the Indian context, adolescents are reliant on their parents usually up until they become adults (after 18 years), which fuels lack of autonomy [31]. Parental restrictions on AST could be due to safety concerns such as those included in the multivariate model (e.g., traffic, dangerous crossings, need to walk through unsafe places), social or cultural norms (e.g., not fashionable), lack of awareness about the importance of AST, or the convenience of dropping their child at school. More than 40% of adolescents reported that their parents did not allow them to walk/cycle to school. These findings underscore the complexity of active school travel and reiterates the need for studies that explore influences comprehensively through models such as the social-ecological model. A better understanding of the reasons behind parental restrictions for AST is required. This may include future qualitative studies or specific studies examining concerns, mediation analyses to explore how and why parents influence adolescents AST, as well as studies to inform intervention strategies to positively influence parents’ perceptions.

Adolescents who perceived their school as being located too far to walk or cycle to had 75% lower odds of walking or cycling to school. Previous studies have also consistently identified distance to school as a major negative influence on AST [7,12,32], including a study of 11–14-year-olds in Hyderabad, India [16]. In India, there are no catchment area policies restricting the choice of school. Hence school selection is mainly up to the parents; thus, children may attend schools with better merit/academic performance that are located far from home. It is, therefore, unsurprising that almost 50% of adolescents considered their school to be too far to walk or cycle to. Opting to attend schools located closer to home or creating catchment areas for schools through policy measures may result in increases in AST. It is also necessary to specifically study adolescents who are residentially located within walking or cycling distance to school to better understand barriers associated with AST [33].

The combined score for India-specific barriers/obstruction to walking within neighbourhoods was positively associated with encouraging AST in model 1, but not in model 2. None of the NEWS and NEWS-Y items or scores were included in the multivariate model because they were not associated with AST in model 1, possibly because they relate to the neighbourhood in general, rather than being specific to AST. Previous research [26,34] shows that it is important to study specific physical activity behaviours with context-specific factors related to these behaviours. For example, examining active school travel with factors relating to the quality and conduciveness of the route taken to reach school.

It should also be noted that the lack of association between built environment features and AST may be because adolescents are not aware of the presence of environmental variables such as traffic safety lights, crossings and other similar neighbourhood features. This may be particularly relevant for minimally active adolescents and among adolescents who were chauffeured between places [30,35], as previous studies have indicated a possible lack of awareness of neighbourhood or environmental features even when adolescents frequently travelled through those places. As half of the adolescents in this study reported that they did not usually walk or cycle to school even once per week, this is a possible underlying factor. It could also be that certain environmental features such as the presence of potholes or stray dogs, while perceived by many to be a barrier, were not associated with AST because they are common, and adolescents may not have experienced travel routes without them.

This study is important, as it is the first to assess a wide range of barriers and neighbourhood-level correlates of active travel to and from school among adolescents in India; however, the limitations of this cross-sectional study should be acknowledged. The prevalence of AST was lower than in a previous study conducted among adolescents in Chennai in 2009, which found 65% walked or cycled to school every day [19]. While rates of AST may have changed over time, it is possible that the current sample may not be representative of the adolescent population of Chennai. Furthermore, the findings are not generalizable to adolescents living in rural or regional areas, since the sample was entirely sourced from a single metropolitan city. Although inclusion of only one metropolitan city could be considered a limitation of the study, it does allow the provision of important contextual information for Chennai and its residents to inform local policy. It is also important to acknowledge that data was collected across an extended period. It is possible that school travel patterns vary over the year according to climatic conditions and this may have impacted our ability to identify associations.

In addition, the self-report measure of AST is prone to recall bias when reporting mode of travel in an average week, and over-reporting due to potential social desirability. Due to the low frequency of AST in the sample, the current study did not examine “regular” AST or trips per week. Similarly, the survey did not capture the reason parents are not supportive of AST.A further limitation is the lack of psychometric testing of the potential correlates in this population group. Although an adapted version has since been shown to have acceptable reliability among adults in India [36], two scales (street connectivity and land use mix access) had poor internal reliability in this study. It will be important for future work to test psychometric properties among adolescents in India. Future studies should explore parents’ fear-based perceptions about safety and social norms and cultural barriers to AST (e.g., acceptable behaviour, parents frowned upon for letting their children walk by themselves). Finally, the study may have been underpowered for the number of independent variables in the multivariate model [37]. Future research should also examine the possibility of harnessing concern about environment to shift travel mode (e.g., air pollution can be reduced through AST) and facilitators of AST (e.g., accompaniment by friends and siblings, availability of green spaces around school, parents able to arrive at work late once or twice a week to help spend more time with their children), which could provide valuable information for interventions. In addition, future studies could examine whether better infrastructure could shift travel behaviour and enhance active school travel. For example, cycle lanes are being constructed in Chennai as part of the smart city initiative [38] and could impact perceived safety and AST.

## 5. Conclusions

Being one of the first studies of this nature in India, this study provides initial insights into ecological correlates of AST among adolescents in India. Only 52% of adolescents used AST at least once per week, and there was inequity in AST based on family income. Therefore, there is considerable scope to improve rates of AST in India. Parental restrictions and distance to school were the strongest barriers to AST in the empirical analyses. Future prospective studies should explore key environmental, social, cultural or individual drivers of parental restriction of AST.

## Figures and Tables

**Table 1:** Adolescents’ self-reported travel mode to or from school (≥1 trip/week)—overall, by gender, age group and income (*n* = 324).

	Active School Travel (%)	Walking (%)	Cycling (%)	Skateboard (%)	Public Transport (%)	Car (%)	Assisted Travel Mode ^4^ (%)
Overall	51.5	32.4	21.9	0.3	25.6	3.7	44.4
Gender ^1^							
Boys	51.8	26.5	28.3	0.6	18.1	3.6	44.6
Girls	51.3	38.6	15.2	0.3	33.5	3.8	44.3
*p*-value	0.922	0.020	0.004	0.329	0.001	0.931	0.962
Age group ^1^							
12–14 years	43.4	25.0	20.3	0.0	24.1	4.2	48.6
15–17 years	67.0	46.4	25.0	0.9	28.6	2.7	36.6
*p*-value	<0.001	<0.001	0.329	0.168	0.376	0.478	0.039
Income ^5^							
Low Income	58.6 *	46.8 *	14.4 *	0.0 *	33.3	0.0 *	25.2 *
Middle Income	54.5	29.4	28.7 *	0.7 *	22.4	3.5	49.0
High Income	33.8 *	16.2 *	19.1	0.0	20.6	8.8 *	66.2 *
*p*-value	0.004	<0.001	0.020	0.534 ^2^	0.077	0.007 ^3^	0.000

^1^ Chi-square tests; ^2^ Fischer exact test since 0 cells have expected count less than 5; ^3^ Fischer exact test since 3 cells have expected count less than 5; ^4^ Any mode of travel where the adolescent is accompanied by another person or on a two wheeler (e.g., parents, friends or other travellers) includes auto rickshaw, cycle rickshaw, school bus, motor bike, car and public transport; ^5^ ANOVA with Scheffé post hoc tests to assess differences between low, middle and high income; * indicates differences (*p* < 0.05) between income categories.

**Table 2:** Reported barriers (%), perceived neighbourhood environment (mean, sd) and odds of reporting active travel to school (≥1 trip/week).

Correlates	Percent (%) or Mean (SD)	Model 1: Active School Travel (Ref: 0 Trips/Week) OR (95% CI) ^1^	Model 2: Active School Travel (Ref: 0 Trips/Week) OR (95% CI) ^2^
Environmental level barriers: Adolescents (Ref: disagree) ^4^	%		
No pavements or cycle lanes	49.4	1.21 (0.73–2.02)	-
Route is boring	43.8	1.07 (0.64–1.77)	-
Route does not have good lighting	28.1	0.97 (0.54–1.76)	-
One or more dangerous crossings	47.5	0.57 (0.34–0.95) *	1.01 (0.54–1.89)
Open drains on the road	28.7	0.99 (0.57–1.73)	-
Potholes/stagnant water on road	43.2	1.24 (0.73–2.09)	-
Roads often shut for renovation	31.9	0.69 (0.40–1.19)	-
Nowhere to leave a cycle safely	21.9	1.15 (0.65–2.04)	-
Too far to walk or cycle	44.8	0.18 (0.09–0.31) *	0.25 (0.13–0.47) *
Too many hills	1.5	0.35 (0.04–2.82)	-
Too much traffic	55.6	0.48 (0.28–0.84) *	0.81 (0.42–1.56)
**Social/family level barriers: Adolescents (Ref: disagree) ^4^**	**%**		
No other teens walk/cycle	27.2	0.60 (0.35–1.03)	-
There are stray dogs	45.7	1.32 (0.77–2.28)	-
Many public meetings on road	26.5	0.69 (0.40–1.18)	-
Would have to walk/cycle through unsafe places (crime)	23.8	0.38 (0.22–0.68) *	0.86 (0.41–1.80)
Not considered fashionable	29.3	0.57 (0.34–0.94) *	0.82 (0.44–1.49)
Parents drive me on way to work	22.8	0.64 (0.36–1.12)	-
Parents do not allow me to walk/cycle?	36.4	0.14 (0.07–0.27) *	0.18 (0.09–0.39) *
**Individual level barriers: Adolescents (Ref: disagree) ^4^**	**%**		
Too much stuff to carry to school	57.1	1.03 (0.60–1.79)	-
Easier to drive or get driven	47.8	0.84 (0.53–1.32)	-
Involves too much planning ahead	36.1	0.97 (0.56–1.68)	-
I get too hot and sweaty	54.6	0.91 (0.52–1.57)	-
Do not enjoy	25.3	0.52 (0.31–0.88) *	0.95 (0.51–1.79)
Too lazy to walk or cycle to school	22.9	0.47 (0.27–0.80) *	0.95 (0.50–1.80)
Not enough time in the morning	43.8	0.61 (0.36–1.02)	-
**Perceptions of crime safety: Adolescents ^5^**	**Mean (SD)**		
Crime safety ^3^	3.08 (1.00)	1.02 (0.82–1.30)	-
Low stranger danger ^3^	3.17 (0.89)	1.20 (0.88–1.64)	-
**Perceptions of crime safety: Parent ^5^**	**Mean (SD)**		
Crime safety ^3^	2.91 (1.08)	1.04 (0.84–1.28)	-
Low stranger danger ^3^	2.87 (0.67)	1.22 (0.84–1.79)	-
**Destinations and neighbourhood features score: Adolescents ^5^**	**Mean (SD)**		
Recreation facilities—Private and Public ^3^	1.66 (0.59)	1.42 (0.95–2.12)	-
Public recreation spaces ^3^	1.92 (0.64)	0.81 (0.54–1.22)	-
**Destinations and neighbourhood features score: Parent ^5^**	**Mean (SD)**		
Land Use Mix—diversity overall ^3^	2.65 (0.53)	1.27 (0.82–1.97)	-
Public recreation spaces ^3^	1.86 (0.66)	0.96 (0.68–1.37)	-
Land use mix—access ^3^	3.41 (0.64)	1.29 (0.88–1.89)	-
India specific barriers to walking ^3^	2.55 (0.77)	1.35 (1.03–1.79) *	1.03 (0.73–1.47)
Street connectivity ^3^	2.85 (0.75)	1.01 (0.77–1.33)	-
Infrastructure for walking and cycling ^3^	1.75 (0.93)	0.78 (0.58–1.03)	-
Aesthetics ^3^	1.66 (0.79)	1.00 (0.74–1.36)	-
Residential density (range: 398–1541) ^3^	564.80 (161.50)	0.99 (0.99–1.00)	-

^1^ Model 1: Logistic regression, adjusted for gender, age, income and mode of recruitment, and accounting for clustering by school; ^2^ Model 2: Multivariate Logistic regression, adjusted for all significant correlates in Model 1, gender, age, income and mode of recruitment, and accounting for clustering by school; ^3^ Composite/average score; higher scores indicate safer perception, activity friendly or more destinations; ^4^ Disagree: proportion responding somewhat disagree or strongly disagree; ^5^ Proportion responding agree or strongly agree; * indicates differences (*p* < 0.05) between income categories.
